# Eiger-induced cell death relies on Rac1-dependent endocytosis

**DOI:** 10.1038/cddis.2016.80

**Published:** 2016-04-07

**Authors:** W Ruan, A Srinivasan, S Lin, k-I Kara, P A Barker

**Affiliations:** 1 Montreal Neurological Institute, McGill University, Montreal, Quebec, Canada; 2 Department of Biology, The University of British Columbia, Okanagan Campus, Kelowna, British Columbia, Canada

## Abstract

Signaling via tumor necrosis factor receptor (TNFR) superfamily members regulates cellular life and death decisions. A subset of mammalian TNFR proteins, most notably the p75 neurotrophin receptor (p75NTR), induces cell death through a pathway that requires activation of c-Jun N-terminal kinases (JNKs). However the receptor-proximal signaling events that mediate this remain unclear. *Drosophila* express a single tumor necrosis factor (TNF) ligand termed Eiger (Egr) that activates JNK-dependent cell death. We have exploited this model to identify phylogenetically conserved signaling events that allow Egr to induce JNK activation and cell death *in vivo*. Here we report that Rac1, a small GTPase, is specifically required in Egr-mediated cell death. *rac1* loss of function blocks Egr-induced cell death, whereas Rac1 overexpression enhances Egr-induced killing. We identify Vav as a GEF for Rac1 in this pathway and demonstrate that dLRRK functions as a negative regulator of Rac1 that normally acts to constrain Egr-induced death. Thus dLRRK loss of function increases Egr-induced cell death in the fly. We further show that Rac1-dependent entry of Egr into early endosomes is a crucial prerequisite for JNK activation and for cell death and show that this entry requires the activity of Rab21 and Rab7. These findings reveal novel regulatory mechanisms that allow Rac1 to contribute to Egr-induced JNK activation and cell death.

Tumor necrosis factor (TNF) is an important cytokine that regulates a variety of cellular process, including proliferation, differentiation, and survival.^1^ Misregulation of its function has been implicated in conditions that range from cancer and autoimmune disease to neurodegenerative disease. Upon engagement of its cognate receptors, it triggers several downstream signaling cascades. The c-Jun N-terminal kinase (JNK) cassette is a key downstream mediator of TNF signaling pathway. Upon activation, JNK is translocated into the nucleus where it phosphorylates and activates activator protein 1 (AP1) and specificity protein 1 transcription factor complexes. These transcription factors then go on to regulate gene expression that can mediate positive or negative effects.^2, 3, 4, 5^

The TNF–JNK signaling pathway is conserved in *Drosophila*. The overexpression of Egr in the eye binds receptors that activate the fly JNK (Basket (Bsk)) through a mitogen-activated protein kinase (MAPK) cascade and thereby induces massive photoreceptor cell (R cell) death, causing a ‘small eye' phenotype.^6, 7, 8, 9^ *Drosophila* genetic tools have been successfully used to dissect the Egr signaling pathway. Many signaling components have been identified in Egr-induced killing, including the cell surface receptors Wengen and Grindelwald and intracellular components such as *Drosophila* TNF receptor-associated factor 2, Bendless and *Drosophila* TAK1-binding protein 2.^10, 11, 12, 13, 14^ This framework provides a powerful system for identifying and characterizing the role of potential signaling components.

In this study, we first demonstrate that Ras-related C3 botulinum toxin substrate 1 (Rac1), a small guanosine triphosphatase (GTPase), has a key role in Egr-induced cell death. We then dissect out the molecular mechanisms of the suppression of Egr-induced killing by knocking down Rac1. We show that Rac1 is required for entry of Egr into early endosomes from which it apparently activates JNK signaling. Altering the expression levels of early endosome protein Ras-related protein 21 (Rab21) or late endosome protein Rab7 has profound effects on Egr-induced cell death. We show that Vav, a guanine nucleotide exchange factor (GEF),^15, 16^ for Rac1 positively regulates Egr-induced killing, whereas dLRRK, a fly homolog of human leucine-rich repeat kinase 2 (LRRK2), functions as a negative regulator of Rac1^17^ to negatively regulate Egr-induced killing. Taken together, our data show that Rac1-dependent production of an Egr signaling endosome is a crucial element required for activation of the cell death pathway in fly.

## Results

### Rac1 positively regulates Egr-induced cell death

**Figure 1:**
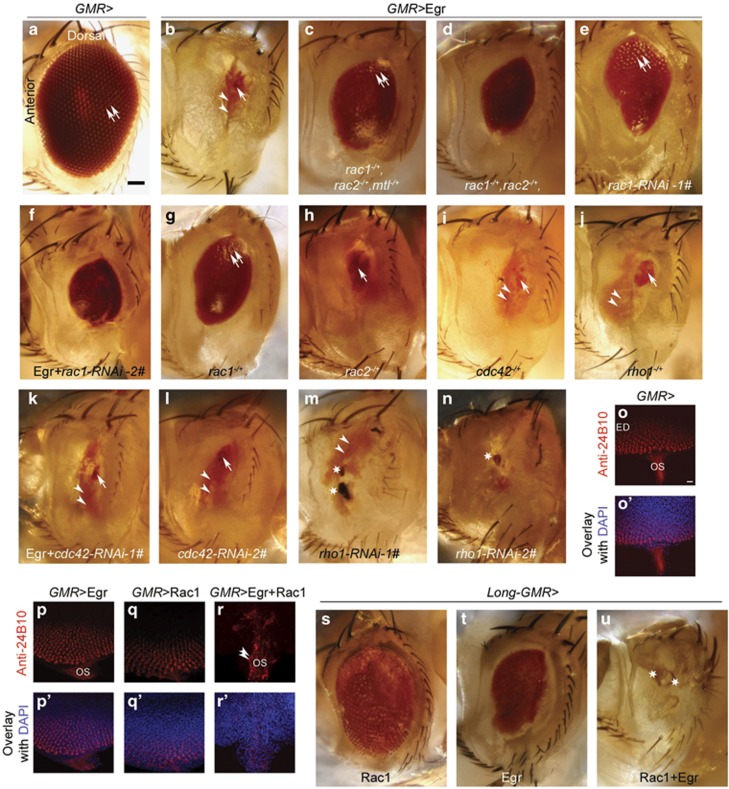
Rac1 is required in *GMR>*Egr-induced cell death pathway. (**a**–**n** and **s**–**u**) Light micrographs of *Drosophila* adult eyes (anterior is to the left and dorsal is up). Double arrows indicates separated ommatidia, arrow indicates the small dot-like red eye tissue, arrow head indicates the yellowish scare-like tissue, and star indicates the brown or black necrosis-like tissue. (**o**–**r** and **o**'–**r**') Maximum projection of staking confocal images of EDs at third instar larvae stage. (**a**) WT (*GMR-Gal4/UAS-sod2*). (**b**) *GMR>*Eg*r* induces cell death resulting in ‘small eye' phenotype (*GMR-Gal4,UAS-egr/+*). (**c**) *GMR>*Egr ‘small eye' phenotype is suppressed by removing one copy of small GTPases, *rac1*, *rac2* and *mtl*, double arrow indicating the rescued ommatidia. (*GMR-Gal4,UAS-egr /+ rac1* ^*J10*^*, rac2* ^*Δ*^*,FRT2A, mtl* ^*Δ*^*/+*). (**d**) *GMR>*Egr ‘small eye' phenotype is suppressed by removing one copy of *rac1* and *rac2* (*GMR-Gal4,UAS-egr /+ rac1* ^*J11*^*, rac2* ^*Δ*^*/+*). (**e** and **f**) Knocking down Rac1 rescues *GMR>*Egr ‘small eye' phenotype (genotypes: In (**e**): *GMR-Gal4,UAS-egr/UAS-rac1-RNAi-1#.* In (**f**): *GMR-Gal4,UAS-egr /+ UAS-rac1-RNAi-2#/+*). (**g**) Removing one copy of *rac1* suppresses *GMR>*Egr ‘small eye' phenotype (*GMR-Gal4,UAS-egr /+ rac1* ^*J11*^*,FRT 2A/+*). (**h**–**j**) *GMR>*Egr ‘small eye' phenotype is not suppressed by removing one copy of *rac2*, (**h**: *GMR-Gal4,UAS-egr/+ rac2* ^*Δ*^*/+*), *cdc42* (**i**: *cdc42* ^*1*^*/+ GMR-Gal4,UAS-egr/+*), or *rho1* (**j**: *GMR-GAL4, UAS-egr/rho1*^*E3.10*^). (**k** and **l**) Knocking down Cdc42 with two different RNAi lines cannot suppress *GMR>*Egr ‘small eye' phenotype (genotypes: In (**k**): *UAS-cdc42-RNAi-1#/+GMR-Gal4,UAS-egr/+.* In (**l**): *GMR-Gal4,UAS-egr/UAS-cdc42-RNAi-2#*). (**m** and **n**) The *GMR>*Egr ‘small eye' phenotype is enhanced to a ‘no eye' phenotype by knocking down two different *rho1* RNAi lines (genotypes: In (**m**): *GMR-Gal4,UAS-egr /UAS-rho1-RNAi-1#*. In (**n**): *GMR-Gal4,UAS-egr /UAS-rho1-RNAi-2#*). (**o**–**r**) EDs were stained with anti-24B10 (red), which specifically recognizes R-cell protein Chaoptin. (**o**'–**r**') Overlay images with DAPI. (**o**–**o**') In WT, at third instar larvae stage each ommatidium was formed with differentiated R-cell clusters. These ommatidia were spaced regularly. R cells send their axons passing through optic stalk (OS) and terminating into brain. The OS is free of R-cell bodies. (**p** and **q**) Regularly patterned ommatidia were observed in overexpression of Egr ((**p** and **p**'): *GMR-Gal4,UAS-egr/+*) or Rac1((**q** and **q**'): *GMR-Gal4/+ UAS-rac1/+*). (**r** and **r**') Overexpression of Egr and Rac1 together disrupted the regularly patterned ommatidia and caused the R cells moving into optic stalk (arrow heads in (**r**): *GMR-Gal4,UAS-egr/+ UAS-rac1/+*). (**s**) *Long-GMR>*Rac1 shows rough eye phenotype (*long-GMR/UAS-rac1w*). (**t**) *Long-GMR>*Egr shows rough and medium-sized eye (*UAS-egr/+ long-GMR/+*). (**u**) *Long-GMR>*Rac1+Egr shows a ‘no eye' phenotype, stars indicating the dark brown necrosis-like tissue (*UAS-egr/+ long-GMR/UAS-rac1W*). Scale bars: 100 *μ*m in (**a**); 10 *μ*m in (**o**)

Overexpression of Egr driven by glass multiple promoter (*GMR*)*-Gal4* driver induces massive cell death in *Drosophila*, resulting in a ‘small eye' phenotype in which flies have a very small dot-like red eye tissue (arrow in Figure 1b) and some yellowish scare-like tissue (arrow heads in Figure 1b) in the eye.^6, 7^ Activation of the JNK cascade has an essential role in the Egr-induced cell death and components lying upstream and downstream of the *Drosophila* JNK homolog (Bsk) have been identified.^9^ Although most mammalian tumor necrosis factor receptor (TNFR) superfamily members do not rely on JNK signaling to induce cell death, JNK-dependent apoptosis is a hallmark of the p75NTR^18, 19, 20^ and its structure is very similar to *Drosophila* TNFR, Wengen. Given this, we have considered whether other signaling events implicated in the mammalian p75NTR cascade are also important for Egr-dependent death in *Drosophila*.

JNK-dependent apoptosis mediated by p75NTR relies on activation of the Rac1 GTPase.^18^ The precise role of Rac1 in the p75NTR cascade remains uncertain, and therefore in this study, our goal was first to determine whether Rac1 lies on the apoptotic pathway induced by Egr and then to establish the function of Rac1 in this pathway. To address this, we crossed a deletion mutant lacking one copy of the three Rac family members in *Drosophila* (*rac1*, *rac2*, and Mig-2-like (*mtl*)) in the *GMR>*Egr overexpression background and then assessed the suppression of the ‘small eye' phenotype. Figures 1a–c show that these flies displayed strong suppression of the *GMR>*Egr ‘small eye' phenotype in which flies have much larger red eye tissues containing individual ommatidia (see double arrows in Figure 1c; penetrance 100% *n*=28). In flies lacking one copy of *rac1* and *rac2*, the *GMR>*Egr ‘small eye' phenotype was still largely suppressed (penetrance 100%, *n*=63; Figure 1d). We further deleted one copy of *rac1* and found that it showed the same suppression of *GMR>*Egr ‘small eye' phenotype as deleting one copy of *rac1* and *rac2* (penetrance 100%, *n*=27; Figure 1g). In contrast, flies lacking one copy of *rac2* did not show suppression of the *GMR>*Egr ‘small eye' phenotype (penetrance 94.3%, *n*=44; Figure 1h). We also tested whether the more distantly related GTPase cell division control protein 42 (Cdc42) and Ras homolog gene family, member 1 (Rho1) are required for *GMR>*Egr-induced cell death. Flies lacking one copy of *cdc42* or *rho1* showed normal *GMR>*Egr ‘small eye' phenotype (Figures 1i and j; penetrance 100% for both, *n*=20 for *cdc42*^1^ and *n*=31 for *rho1*^E3.10^). When knocking down Cdc42 with two different RNA interference (RNAi) lines, the ‘small eye' phenotype is not changed, indicating that *cdc42* is not required for this pathway (Figures 1k and l, penetrance 100% for both, *n*=52 for line 1#, *n*=46 for line 2#). Interestingly, knocking down Rho1 with two different RNAi lines produces flies with a dramatic reduction in eye volume. We describe this phenotypes as a ‘no eye' phenotype in which the small dot-like red eye tissue is gone. Instead, flies have some yellowish scare-like (arrow heads in Figure 1m) and black necrosis-like tissues (stars in Figures 1m and n), indicating that Rho1 negatively regulates *GMR>*Egr ‘small eye' phenotype (penetrance 100% for both, *n*=37 for line 1#, *n*=47 for line 2#). Finally, to confirm a specific role for Rac1 in Egr-induced apoptosis, we examined the consequence of Rac1 protein depletion using two separate RNAi lines. Figures 1e and f show that line1# produced strong suppression (penetrance 100%, *n*=52) and line2# produced moderate suppression (penetrance 41%, *n*=34). Taken together, these loss-of-function data indicate that Rac1 has a specific and essential role in Egr-induced R-cell death and Rho1 negatively regulates Egr-induced R-cell death.

Next we performed gain-of-function experiments to determine whether Rac1 activity can enhance Egr signaling. Previous studies have shown that Rac1 overexpression causes a ‘rough eye' phenotype that reflects misregulation of the actin cytoskeleton.^21^ However, when we combined *GMR-Gal4*-driven overexpression of Egr with Rac1, flies died at the yellow pupal stage indicating the potentiation of Egr-induced cell death by Rac1 overexpression. We then checked the eye imaginal disc (ED) at third instar larvae stage; at that stage the ED starts to give rise to the adult eye. When *GMR* is overexpressing Egr or Rac1 alone, R-cell patterning is normal, and the ommatidia are regularly spaced (compare Figures 1o–q). However, *GMR* is overexpressing Egr and Rac1 together, the regularly spaced ommatidia are completely disrupted (compare Figures 1o–r) and the R cells moved into optic stalk (double arrow head in Figure 1r), further indicating that the overexpressing Egr can potentiate Rac1 function.

To overcome the lethality caused by *GMR* driver, we used the *long-GMR-Gal4*, a weaker and more eye-specific driver, to overexpress Egr with Rac1. The *long-GMR-Gal4*-driven overexpression of Egr, or Rac1, caused a ‘rough eye' phenotype (penetrance 100% for both; Figure 1s, *n*=76 and see our previous work;^17^ Figure 1t, *n*=25). When Rac1 and Egr were co-expressed by *long-GMR-Gal4*, flies display a ‘no eye' phenotype in which the small dot-like red eye tissue is gone. Instead, flies have brown necrosis-like tissue in the eye (stars in Figure 1u; penetrance 100%, *n*=31).

### Overexpression of Egr causes activation of Rac1 and JNK *in vivo*

**Figure 2:**
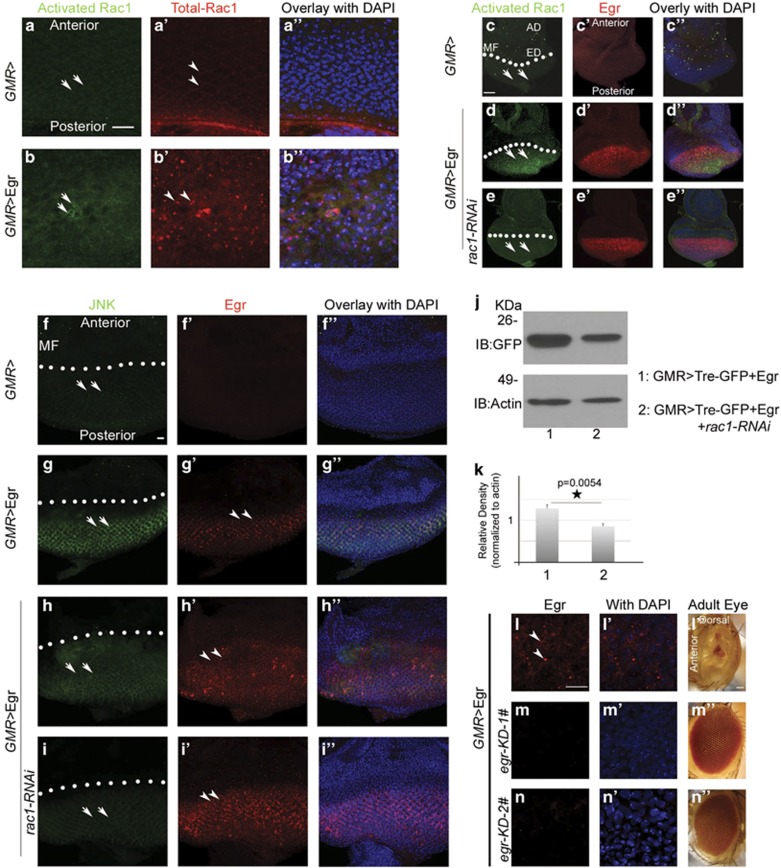
Rac1 and JNK are activated in response to overexpression of Egr. EDs of third instar larvae. The fluorescence images were taken under same detector gains and at same focal planes for comparing the expression levels (anterior is up). The white dotted lines in (**c**–**i**) denote the MF. *GMR* drives proteins to be expressed in the region posterior to the MF. (**a**–**e**) GFP signals indicate activated GTP-Rac1 proteins. Images in (**c**-**e**), (**c**'-**e**'), and (**c**''–**e**'') were taken at the lower magnification, which showed the whole ED and partial antenna imaginal disc (AD). (**a**'–**b**') Anti-Rac1 staining shows total Rac1 proteins. (**a**''–**b**'') Overlay images with DAPI. (**c**'–**e**') Anti-Egr staining. (**c**''–**e**'') Overlay images with DAPI. (**a**–**a**'') In WT ED (*GMR-Gal4/sqh-PAK1-RBD-GFP*), weakly activated GFP-Rac1 proteins are shown (**a**) and total Rac1 proteins are located on the cell membrane (arrow heads in **a**'). (**b**–**b**'' and **d**–**d**'') EDs of *GMR>*Egr (*GMR-Gal4,UAS-egr/sqh-PAK1-RBD-GFP*). GTP-Rac1 proteins are largely increased in response to Egr overexpression in the region posterior to MF (arrows in **b** and **d**) and total Rac1 proteins are also increased and form puncta-like structures (arrow heads in **b**'). (**c**–**c**'') WT (*GMR-Gal4/sqh-PAK1-RBD-GFP*). (**e**–**e**'') In response to overexpression of Egr, activated GFP-Rac1 is decreased when Rac1 is knocked down (compare arrows in **c**–**e**). (genotypes: In (**e**–**e**''): *GMR-Gal4,UAS-egr,UAS-rac1-RNAi-1#/sqh-PAK1-RBD-GFP*). (**f**–**i**) Anti-GFP staining shows JNK activation. (**f**'–**i**') Anti-Egr staining shows Egr proteins. (**f**''–**i**'') Overlay images with DAPI. (**f**) In WT ED (*GMR-Gal4/TRE-GFP-16*), JNK is weakly activated (arrows in **f**). (**g**–**g**'') EDs of overexpressing *UAS-egr* transgene (*GMR-Gal4,UAS-egr/TRE-GFP-16*). (**g**) JNK is strongly activated in the region posterior to MF (arrows in **g**). (**g**') Egr proteins are expressed in the eye (arrow heads in **g**'). (**h**–**h**'') Overexpression Egr (*GMR-Gal4,UAS-egr/TRE-GFP-16*). (**i**–**i**'') Knocking down Rac1 in *GMR>*Egr background (*GMR-Gal4,UAS-egr*, *UAS-rac1-RNAi-1#/TRE-GFP-16*). The activated JNK signal is reduced when Rac1 is knocked down (compare **h** and **i**) but the Egr signal is unchanged (compare **h**' and **i**'). (**j** and **k**) Western blotting analysis. (**j**) Each lane was loaded with lysates made from one eye–brain complex. (**k**) Quantification of four independent experiments. (Student's *T-*Test, paired, two tails; *P=0.0045). (**l**–**l**'') Overexpression Egr (*GMR-Gal4,UAS-egr/+*). Egr protein is detected in the ED (arrow heads in **l**). (**m**–**m**'') and (**n**–**n**'') knocking down Egr with two different *egr-RNAi* lines in *GMR>*Egr background. (genotypes: In (**m**–**m**''): *GMR-Gal4,UAS-egr/UAS-egr-RNAi-1#*. In (**n**–**n**''): *GMR-Gal4,UAS-egr/UAS-egr-RNAi-2#*). Scale bars in (**a**), (**f**), and (**l**): 10 *μ*m; in (**c**): 50 *μ*m; in (**l**''): 100 *μ*m

To further determine whether Rac1 GTPase activity is actually activated by Egr, we employed the *sqh-PAK1*^*RBD*^*-GFP* fly line to monitor Rac1 activation *in vivo*.^22^ In this line, PAK1^RBD^-GFP (green fluorescent protein) protein produced from a myosin promoter (spaghetti squash (sqh)) is detected only when it bound to activated Rac1. Anti-GFP immunostaining on EDs of *GMR>*Egr or *GMR* flies bearing this transgene revealed a dramatically enhanced PAK1^RBD^-GFP signal in the region after the morphogenetic furrow (MF) in *GMR>*Egr EDs (arrows in Figures 2b and d) compared with *GMR* controls in which there is no enhanced PAK1^RBD^-GFP signal in the region after MF (arrows in Figures 2a and c) at the third instar larval stage. The increased GFP signal is reduced when Rac1 is knocked down (compare arrows in Figures 2c–e). These results suggest that the Egr overexpression can activate endogenous Rac1 *in vivo*. Interestingly, levels of total Rac1 are enhanced as well (compare arrow heads in Figures 2a' and b'), suggesting that Egr signaling may lead to Rac1 stabilization or increase the transcription of *rac1*.

We next asked whether the JNK activation induced by Egr occurs through a Rac1-dependent pathway *in vivo*. For this, we employed *TRE-GFP*, a line in which GFP expression is controlled by a JNK-responsive TPA or tetradecanoylphorbol acetate response element (TRE).^23^ Figures 2f and g show that GFP levels are barely detectable in *GMR* control line, whereas Egr overexpression results in a large increase in GFP signal indicating the strong JNK activation (arrows in Figure 2g). However, when Rac1 levels were depleted using RNAi, Egr-induced GFP signal was decreased (compare Figures 2h and i), indicating that Rac1 activation is indeed required for Egr-induced JNK activation *in vivo*. Western blotting analysis of the EDs also shows that GFP levels in the eyes of Rac1 depletion by RNAi was decreased (compare lanes 1 and 2 in Figures 2j and k).

One potential caveat of this approach is that Rac1 depletion may somehow suppress Egr expression. To address this, we produced an anti-Egr antibody and used this to determine whether Egr is equivalently expressed in the presence and absence of Rac1. Figures 2l–n show anti-Egr immunostaining of *GMR>*Egr EDs at third instar larval stage in the absence or presence of Egr RNAi knockdown. Egr accumulation was clearly present in the *GMR>*Egr imaginal disks (arrow heads in Figure 2l) and this was sharply depleted in each of the lines expressing distinct forms of Egr RNAi (Figures 2m and n), demonstrating that this antibody can specifically detect Egr expression *in vivo*. Furthermore, knocking down Egr with these two RNAi lines completely reverts the *GMR>*Egr ‘small eye' phenotype (Figures 2m'' and n''). We then used immunocytochemistry to determine whether Egr levels were altered in the absence and presence of Rac1 knockdown. Figures 2h' and i' show that Egr levels are readily detectable in EDs expressing normal or depleted Rac1. We conclude that Rac1 has a crucial role linking Egr-dependent receptor activation to JNK activity.

### Rac1 regulates Egr endosome localization and the *GMR>*Egr ‘small eye' phenotype is modulated by changing levels of early or late endosome Rab proteins

**Figure 3:**
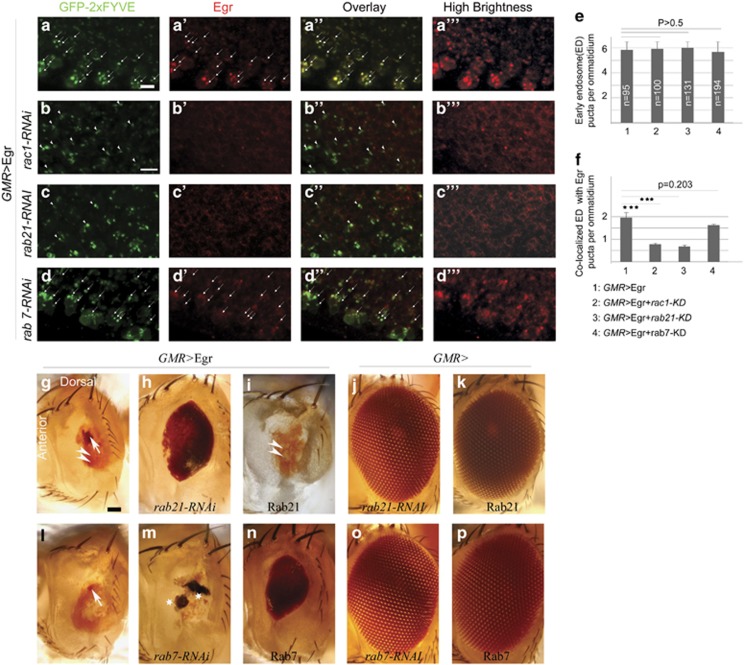
Egr proteins are localized in early endosomes and changing the protein levels of Rabs modifies the *GMR>*Egr ‘small eye' phenotype. (**a**–**a**''', **b**–**b**''', **c**–**c**'', and **d**–**d**''') EDs of third instar larvae (anterior is up). (**a**–**d**) Anti-GFP staining shows early endosomes. (**a**'–**d**') Anti-Egr staining. (**a**''–**d**'') Overlay images. (**a**'''–**d**''') High brightness/contrast images of **a**'–**d**'. (**a**–**a**''') EDs of *GMR>*Egr (*GMR-Gal4,UAS-egr/UAS-GFP-myc-2xFYVE*). Many Egr proteins are in puncta-like structures (arrows in **a**') that were co-localized with GFP-2XFYVE, which labels early endosomes (arrows in **a**–**a**''). (**b**–**b**''') EDs of *GMR>*Egr with knocking down Rac1 (*GMR-Gal4,UAS-egr,UAS-rac1-RNAi-1#/UAS-GFP-myc-2xFYVE*). The early endosome localization of Egr proteins is largely reduced by knocking down Rac1 (arrow heads in **b** and **b**''). (**c**–**c**''') EDs of *GMR>*Egr with knocking down Rab21 (*GMR-Gal4,UAS-egr/UAS-GFP-myc-2xFYVE; UAS-rab21-RNAi/+*). The early endosome localization of Egr proteins is largely reduced by knocking down Rab21 (arrow heads in **c** and **c**''). (**d**–**d**''') EDs of *GMR>*Egr with knocking down Rab7 (*GMR-Gal4,UAS-egr/UAS-GFP-myc-2xFYVE; UAS-rab7-RNAi/+*). The early endosome localization of Egr proteins is not changed by knocking down Rab7 (arrows in **d** and **d**''). (**e** and **f**) Quantifications of the number of early endosomes (**e**) and co-localization of Egr with early endosomes (**f**). The number of early endosomes per ommatidium is same among different genotypes, whereas knocking down Rac1 or Rab21 significantly reduced Egr early endosome localization (***P<0.001, Student's *T*-test, paired, two tails). (**g**–**p**) Light micrographs of *Drosophila* adult eyes (anterior is to the left and dorsal is up). Arrow indicates the small dot-like red eye tissue, arrow head indicates the yellowish scare-like tissue, and star indicates the black necrosis-like tissue. (**g**) *GMR>*Eg*r* (*GMR-Gal4,UAS-egr/+*). (**h** and **i**) The *GMR>*Egr ‘small eye' phenotype is suppressed by knocking down Rab21 (**h**) and enhanced by overexpressing Rab21 (**i**). (genotypes: In (**h**): *GMR-Gal4,UAS-egr/+ UAS-rab21-RNAi/+*. In (**i**): *GMR-Gal4,UAS-egr/UAS-YFP-rab21*). (**j**) WT eye (*GMR-Gal4/+ UAS-rab21-RNAi/+*). (**k**) WT eye (*GMR-Gal4/UAS-YFP-rab21*). (**l**) *GMR>*Egr (*GMR-Gal4,UAS-egr/+*). (**m** and **n**) The *GMR>*Egr ‘small eye' phenotype is enhanced by knocking down Rab7 (**m**) and suppressed by overexpression of Rab7 (**n**). (genotypes: In (**m**): *GMR-Gal4,UAS-egr/+ UAS-rab7-RNAi/+*. In (**n**): *GMR-Gal4,UAS-egr/+ UAS-rab7-GFP/+*). (**o**) WT eye (*GMR-Gal4/+ UAS-rab7-RNAi/+*). (**p**) WT eye (*GMR-Gal4/+ UAS-rab7-GFP/+*). Scale bars in (**a**) and (**b**): 5 *μ*m; in (**g**): 100 *μ*m

Previous studies of the *GMR>*Egr-induced ‘small eye' phenotype suggest that JNK activation occurs in the early endosomes.^24^ As Rac1 has been implicated in the initiation of macropinocytosis and Rac1-dependent actin remodelling is required to form endocytotic vesicles, we addressed the possibility that Rac1 is required for Egr entry into early endosome.^25, 26, 27^ We first asked whether Egr is present within these vesicles, using an upstream activating sequence (*UAS*)*-GFP-2XFYVE* transgene to allow identification of early endosomes in *GMR>*Egr flies. Figure 3a–a''' show that Egr protein is clearly co-localized with GFP-2XFYVE, indicating that the ligand does accumulate in this compartment *in vivo*. Depletion of Rac1 has no effect on GFP-2XFYVE localization but dramatically reduces co-localization of Egr with GFP-2XFYVE (Figures 3b–b'''), consistent with the hypothesis that Rac1 activity is required for Egr accumulation in early endosomes. The co-localization of Egr with GFP-2XFYVE was sharply reduced by knocking down early endosome protein Rab21 (Figures 3c–c'''), whereas the co-localization is not changed by knocking down late endosome protein Rab7 (Figures 3d–d''').

To explore the notion that Rac1-dependent early endosome formation is important for Egr signaling, we performed gain- and loss-of-function experiments to manipulate the function of Rab7 and Rab21. Rab7 and Rab21 are small GTPases that have important roles orchestrating movement of intracellular vesicles.^28, 29, 30^ Figure 3h shows that knocking down the early endosome protein Rab21 reduced the *GMR>*Egr ‘small eye' phenotype (penetrance 84.2%, *n*=19; Figure 3h), whereas overexpressing Rab21 enhanced the *GMR>*Egr ‘small eye' phenotype to a ‘no eye' phenotype (penetrance 100%, *n*=19; Figure 3i). In contrast, knockdown of the late endosome protein Rab7 enhanced the *GMR>*Egr ‘small eye' phenotype to a ‘no eye' phenotype (penetrance 100%, *n*=51; Figure 3m) and Rab7 overexpression suppressed the ‘small eye' phenotype (penetrance 99.1%, *n*=67; Figure 3n). Although the co-localization of Egr with GFP-2XFYVEGFP is not changed by knocking down late endosome protein Rab7, the enhancement of Egr ‘small eye' phenotype may reflect persistence of an Egr signaling endosome in the absence of Rab7. Thus manipulations that increase Egr levels in early endosome pool enhance the ‘small eye' phenotype and those that reduce Egr protein suppress it, supporting the hypothesis that the early endosome functions as a crucial hub for Egr signaling.

### Vav regulates Egr-dependent Rac1 activity

**Figure 4:**
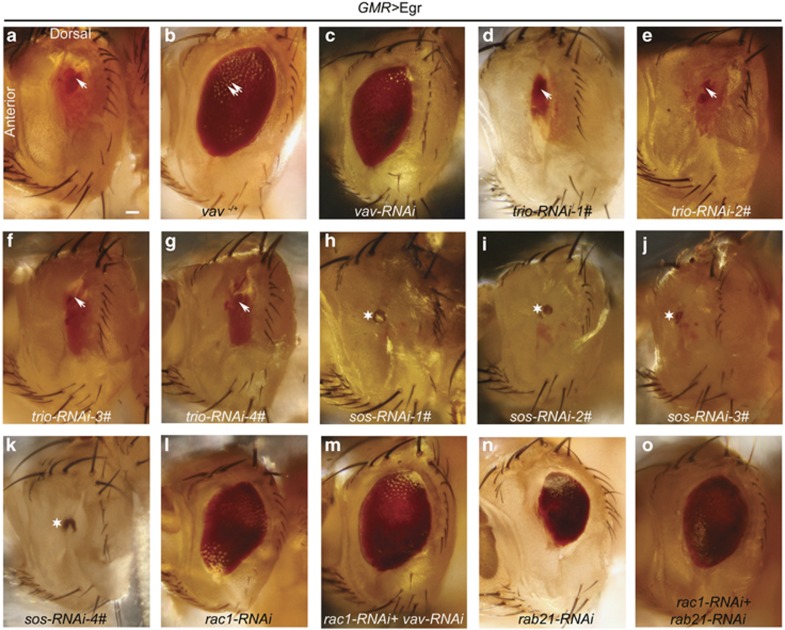
Vav regulates Egr-dependent Rac1 activity. Light micrographs of *Drosophila* adult eyes (anterior is to the left and dorsal is up). Arrow indicates the small dot-like red eye tissue, double arrows indicate separated ommatidia, and star indicates the black necrosis-like tissue. (**a**) *GMR>*Egr (*GMR-Gal4,UAS-egr/+*). (**b** and **c**) Removing one copy of *vav* or knocking down Vav suppressed *GMR>*Egr ‘small eye' phenotype (genotypes: In (**b**): *vav* ^*KG02029*^*/+ GMR-Gal4,UAS-egr/+*. In (**c**): *GMR-Gal4,UAS-egr/+ UAS-vav-RNAi/+*). (**d**–**g**) Knocking down Trio with four different RNAi lines has no effects on *GMR>*Egr ‘small eye' phenotype (genotypes: In (**d**): *GMR-Gal4,UAS-egr/+ tiro-RNAi-1#/+*. In (**e**): *GMR-Gal4,UAS-egr/trio-RNAi-2#*. In (**f**): *GMR-Gal4,UAS-egr/+ trio-RNAi-3#*/+. In (**g**): *GMR-Gal4,UAS-egr/+ trio-RNAi-4#*/+).). (**h**–**k**) Knocking down Sos with four different RNAi lines enhanced *GMR>*Egr ‘small eye' phenotype and showed a ‘no eye' phenotype (genotypes: In (**h**): *GMR-Gal4,UAS-egr/sos-RNAi-1#*. In (**i**): *GMR-Gal4,UAS-egr/sos-RNAi-2#.* In (**j**): *GMR-Gal4,UAS-egr/+ sos-RNAi-3#/+.* In (**k**): *GMR-Gal4,UAS-egr/+ sos-RNAi-4#/+*). (**l**) Rac1 knocking down (*GMR-Gal4,UAS-egr,UAS-rac1-RNAi-1#/+*). (**m**) The knocking down Rac1 or Vav caused suppression of *GMR>*Egr ‘small eye' phenotype is not further enhanced by double knocking down Rac1 and Vav (*GMR-Gal4,UAS-egr,UAS-rac1-RNAi-1#/+ UAS-vav-RNAi/+*) (compare **c**, **l**, and **m**). (**n**) Rab21 knocking down (*GMR-Gal4,UAS-egr/+ UAS-rab21-RNAi/+)*. (**o**) Double knocking down Rac1 and Rab21 cannot further enhance the suppressed *GMR>*Egr ‘small eye' phenotype with single knocking down Rac1 or Rab21 (*GMR-Gal4,UAS-egr,UAS-rac1-RNAi-1#/+ UAS-rab21-RNAi/+*). Scale bar: 100 *μ*m

We next took a candidate gene approach to identify the Rac1 GEF that functions in the *GMR>*Egr signaling pathway. We examined three Rac1 GEFs (Trio, Sos, and Vav) and found that removing one copy of *vav* strongly suppressed the *GMR>*Egr ‘small eye' phenotype (penetrance 87.9%, *n*=33; Figure 4b). Suppression of Egr killing was also observed when Vav expression was suppressed by a *vav-RNAi* line (penetrance 65.6%, *n*=32; Figure 4c). In contrast, knocking down Trio had no effect (penetrance 100% for four lines; Figure 4d, *n*=19; Figure 4e, *n*=13; Figure 4f, *n*=20; Figure 4g, *n*=54), and Sos depletion resulted in a ‘no eye' phenotype (penetrance 100% for four lines; Figure 4h, *n*=23; Figure 4i, *n*=35; Figure 4j, *n*=35; Figure 4k, *n*=87). Furthermore, the suppression of the ‘small eye' phenotype observed with Vav and Rac1 or Rab21 and Rac1 double depletion were not more severe than depleting any one of them, indicating that Vav, Rab21, and Rac1 function on the same pathway (penetrance 100% for both, Figure 4m, *n*=55; Figure 4o, *n*=81).

Together, these results indicate that Vav functions as a Rac1 GEF in the *GMR>*Egr signaling pathway and suggest that Sos negatively regulates this pathway via an unknown mechanism(s).

### dLRRK negatively regulates Egr-dependent Rac1 activity

**Figure 5:**
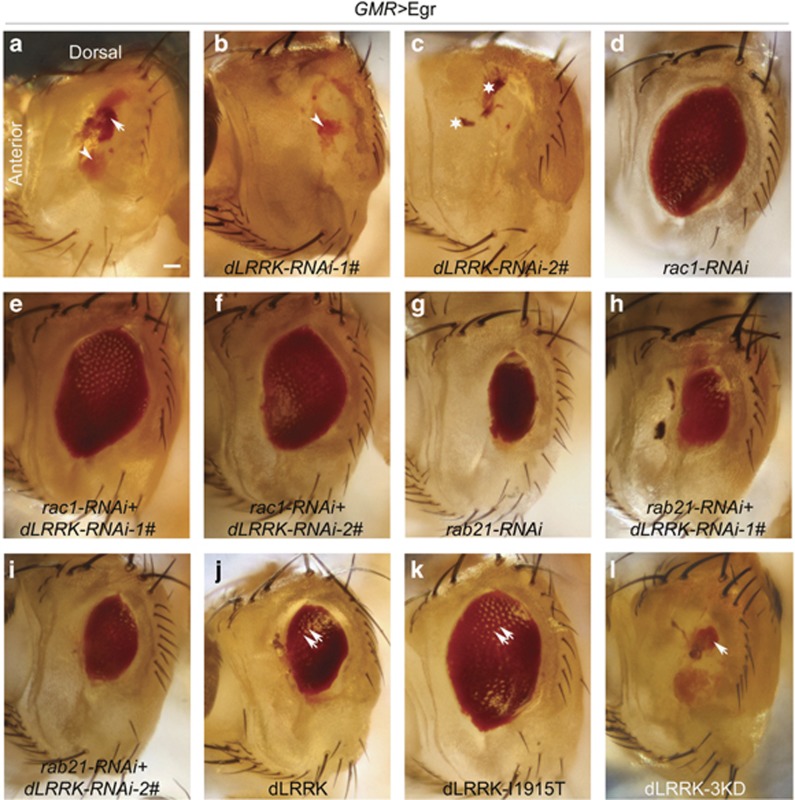
dLRRK negatively regulates Egr-dependent Rac1 activity. Arrow indicates the small dot-like red eye tissue, arrow head indicates the yellowish scare-like tissue, star indicates the brown or black necrosis-like tissue, and double arrows indicate separated ommatidia. (**a**) Overexpression Egr (*GMR-Gal4,UAS-egr/+*). (**b** and **c**) The *GMR>*Egr ‘small eye' phenotype is enhanced by knocking down dLRRK. (genotypes: In (**b**): *GMR-Gal4,UAS-egr/+ UAS-dLRRK-RNAi-1#/+*. In (**c**): *GMR-Gal4,UAS-egr/+ UAS-dLRRK-RNAi-2#/+*). (**d**) Rac1 knocking down (*GMR-Gal4,UAS-egr,UAS-rac1-RNAi-line1#/+).* (**e** and **f**) The negative regulation of *GMR>*Egr with knocking down dLRRK is lost when Rac1 is simultaneously knocked down (genotypes: In (**e**): *GMR-Gal4,UAS-egr,UAS-rac1-RNAi-1#/+ UAS-dLRRK-RNAi-1#/+.* In (**f**): *GMR-Gal4,UAS-egr,UAS-Rac1-RNAi-1#/+ UAS-dLRRK-RNAi-2#/+*). (**g**) Rab21 Knocking down (*GMR-Gal4,UAS-egr/+ UAS-rab21-RNAi/+*). (**h** and **i**) Knocking down dLRRK cannot enhance the *GMR>*Egr ‘small eye' phenotype when Rab21 is simultaneously knocked down (genotypes: In (**h**): *GMR-Gal4,UAS-egr/+ UAS-rab21-RNAi/UAS-dLRRK-RNAi-1#.* In (**i**): *GMR-Gal4,UAS-egr/+ UAS-rab21-RNAi/UAS-dLRRK-RNAi 2#*). (**j**–**l**) *GMR>*Egr ‘small eye phenotype is suppressed by overexpression of a WT dLRRK (j) or a kinase-active form of dLRRK (**k**) but not a kinase-dead form of dLRRK (**l**). (genotypes: In (**j**): *GMR-Gal4,UAS-egr/+ UAS-dLRRK-wt/+*. In (**k**): *GMR-Gal4,UAS-egr/+ UAS-dLRRK* ^I1915T^*/+*. In (**l**): *GMR-Gal4,UAS-egr/+ UAS-dLRRK* ^3KD^/+). Scale bar: 100 *μ*m

We and our colleagues have recently shown that dLRRK and mammalian LRRK2 suppress Rac1 activity in the fly eyes and mammalian cell lines, respectively.^17^ Here we tested whether dLRRK has an impact on Rac1 activity that is required for Egr-induced killing. Figures 5a–c show that the *GMR>*Egr ‘small eye' phenotype was sharply changed to a ‘no eye' phenotype when dLRRK expression was suppressed using either of two distinct RNAi lines (penetrance 72.7%, *n*=154, Figure 5b; penetrance 95.3%, *n*=65, Figure 5c), suggesting that dLRRK normally suppresses Rac1 activity in the Egr-containing early endosome. Consistent with this, cell death increased by dLRRK depletion did not occur in *GMR>*Egr flies in which Rac1 or Rab21 proteins were knocked down (penetrance 89.7%, *n*=108, Figure 5e; penetrance 100% *n*=82, Figure 5f; penetrance 100%, *n*=54, Figure 5h; penetrance 94.5%, *n*=55, Figure 5i). These results suggest that overexpression of wild-type (WT) or constitutively active dLRRK might suppress Egr-dependent R-cell death. Indeed, Figures 5j–l show that overexpression of WT dLRRK significantly rescued the Egr-dependent cell death (penetrance 67.1% *n*=169, Figure 5j) and that overexpression of constitutively active dLRRK (*dLRRK*^*I1915T*^) almost completely blocked the *GMR>*Egr-induced cell death (penetrance 100% *n*=220, Figure 5k). In contrast, flies overexpressing a kinase-dead form of dLRRK (*dLRRK*^3KD^) produced very slightly rescue in the size of the eye in 71.2% flies and none at all in the remaining 28.8% (Figure 5l, *n*=59). These results indicate that the Egr-pathway suppression conferred by dLRRK required its intrinsic kinase activity.

## Discussion

In this study, we show that Rac1 has a critical role in Egr-induced cell death *in vivo*. Previous studies in mammalian systems have shown that Rac1 is activated in response to activation of p75NTR and that this activation causes JNK activation and apoptosis in cell lines.^18^ Here we show that Rac1 is activated in response to overexpression of Egr and this activation is important for JNK activation and cell death *in vivo*. We identify Vav as a GEF for Rac1, and show that dLRRK functions as a negative regulator of Rac1 during Egr signaling. Further, we demonstrate that depletion of the early endosome protein Rab21 reduces, and the late endosome regulator Rab7 enhances, Egr-induced cell death. Taken together, these data support the hypothesis that Egr-induced Rac1 activation has a critical role in regulating the formation of signaling endosomes that are required for subsequent JNK activation and cell death.

Rac1 is a member of Rho family small GTPase that functions as a molecular switch in the regulation of many aspects of cellular behavior, including cell–cell adhesion, cell migration, cell cycle progression, and cellular transformation.^31, 32^ Rac1 has also been implicated in cell death, specifically in the action of pro-apoptotic members of the TNFR superfamily, such as p75NTR, death receptor 4 (DR4) and DR5. With regard to DR4 and DR5, Rac1 has been implicated in activation of NADPH oxidase (NOX) complexes and subsequently generation of reactive oxygen species (ROS).^33^ Rac1 has long been known to act as allosteric regulator of NOX complexes in phagocytes.^34^ Kim *et al.*^35^ have shown that TNF/TNFR1 generates ROS through NOX complex in mouse fibroblasts upon TNF treatment. We tested whether the Rac1-mediated Egr killing involves NOX-dependent ROS generation by knocking down each of the NOX subunits but did not observe any suppression of the Egr-induced killing (data not shown). We conclude that suppression of Egr-induced killing by Rac1 depletion is not due to alterations in NOX function. Previous studies have shown that Egr protein present in early endosomes is important for downstream JNK activation.^24^ As Rac1 activity was previously implicated in endocytosis,^25, 26, 27^ we tested whether Rac1 is required for the localization of Egr in early endosomes. Consistent with this, we found that knockdown of Rac1 blocked the accumulation of Egr in early endosomes.

Rabs are members of Ras-associate binding family of small GTPases that function in discrete steps in endocytosis and vesicular transport.^36^ Rab21 has a crucial role in the initial trafficking events that generate early endosomes, whereas Rab7 contributes to the sorting events that produce late endosomes and drive them to multivesicular bodies/lysosomes for degradation. The substantial reduction of Egr early endosome localization, together with subsequently blocking Egr-induced cell death in flies lacking Rab21, suggests that the Egr signaling complex is assembled on endosomes only after the ligand–receptor assembly is internalized. Conversely, flies lacking Rab7 show a dramatic increase in Egr-induced cell death that likely reflects a defect in the downregulation of the Egr endosomal signaling complex.

By comparing the effects of loss of function of three Rac1 GEFs, we identified the single *vav* gene in *Drosophila* as a crucial GEF required for Egr-induced Rac1 activation. Mammals have three *vav* genes, and in each of these, phosphorylation of Vav at tyrosine residue 174 (Y174) relieves an intramolecular inhibitory interaction that allows the GEF activity to be unleashed. This phosphorylation, and subsequent GEF activity, is enhanced by binding PtdIns (3,4,5) P3 to the PH domain within Vav1.^37, 38^ The domain structures of mammalian and *Drosophila* Vav are identical and the tyrosine residue in *Drosophila* Vav is embedded in a well-conserved motif, and it therefore seems likely that regulatory mechanisms that impinge on *Drosophila* Vav will be similarly conserved. Addressing the importance of the Y174 analog, the upstream kinases that target it, and the role of phosphoinositide lipids in Vav regulation will be interesting topics for future studies.

Mutations in LRRK2 are a major cause of inherited and sporadic forms of Parkinson's disease.^39^ We and our colleagues recently showed that LRRK2 is localized within the endosomal compartment and that it negatively regulates Rac1 activity in mammalian cell lines and the fly eyes.^17^ Several studies have shown the interaction between LRRK2 and signaling components of p38 MAPK and JNK pathways, but the specific links between them are lacking.^40, 41, 42^ Here we found that dLRRK kinase activity acts as a negative regulator of Egr signaling that lies upstream of Rac1 and Rab21. Taken together with earlier findings, these data indicate that dLRRK normally acts to negatively regulate Rac1 activation and thereby temper Egr-induced signaling.

In summary, we have dissected mechanisms supporting Egr-induced cell death. Rac1, Vav, and dLRRK, together with Rab21 and Rab7, coordinately regulate Egr compartmentalization required for cell death.

## Material and Methods

### Drosophila stocks

W1118, GMR-Gal4(9146), long-GMR-Gal4(8121), UAS-rac1W(28874), rac1^J11^ (6674), rac1^J11^, rac2^Δ^(6677), rac1^J10^, rac2^Δ,^, mtl^Δ,^(6679), cdc42^1^(7337), Rho1^E3.10^(3176), UAS-rac1-RNAi(34910), UAS-YFP-rab21(23241), UAS-rab21-RNAi(29403), UAS-rab7-GFP(42706), UAS-rab7-RNAi(27051), vav^KG02022^(14176), UAS-cdc42-RNAi-1#(29004), UAS-cdc42-RNAi-2#(37477), UAS-Rho1-RNAi-1#(9909), UAS-Rho1-RNAi-2#(29002), UAS-trio-RNAi-3#(27732), UAS-trio-RNAI-4#(43549), UAS-sos-RNAi-3#(31275), UAS-sos-RNAi-4#(34833), and UAS-vav-RNAi(39059) lines were provided by Bloomington *Drosophila* Stock Center, Bloomington, IN, USA. UAS-rac1-RNAi (v49246), UAS-egr-RNAI-1#(v45252), UAS-egr-RNAi-2#(v45253), UAS-trio-RNAi-1#(v40137), UAS-trio-RNAi-2#(v40138), UAS-sos-RNAi-1#(v42848), UAS-sos-RNAi-2#(v106925), UAS-dLRRK-RNAi-1#(v22139), and UAS-dLRRK-RNAi-2#(v22140) were provided by Vienna *Drosophila* RNAi Center, Vienna, Austria. UAS-egr was provided by Dr. Konrad Basler.^6^ sqh-PAK1^RBD^-GFP was provided by Dr. Susan Parkhurst.^22^ TRE-GFP was provided by Dr. Dirk Bohmann.^23^ UAS-GFP-myc-2xFYVE was provided by Dr. Hugo Bellen (Baylor College of Medicine, Houston, TX, USA). UAS-dLRRK-WT, UAS-dLRRK-I1915T, and UAS-dLRRK-3KD were provided by Dr. Bingwei Lu.^43^

### Image of fly eyes

Flies were raised on standard fly food containing yeast, corn syrup, and cornmeal at 25 °C. Fly eye pictures were acquired with a Canon EOS 1000D DSLR (rebel XS) (Canon Canada Inc., Montreal, Canada) camera mounted on a Zeiss Axioskop 40 microscope with × 10 objective (0.25=NA) (Carl Zeiss Canada Ltd., Toronto, Canada). In order to obtain pictures of the entire eye in focus, we took a series of pictures at different focal planes of the eye (10–30 pictures depending on the eye shape). Focus stacking was performed on the picture stack with the use of the Helicon Focus software (HeliconSoft, Helicon Soft Ltd., Kharkiv, Ukraine) to generate the final pictures with extended depth of field.

### Plasmid constructs, protein purification, and antibody production

Egr c-DNA (LP03784) was obtained from the *Drosophila* Genomics Resource Center, Bloomington, IN, USA. pMal-C2X (New England Biolab, Whitby, Ontario, Canada) and pGEX-4T-1 (GE, Mississauga, Ontario, Canada) were used to express MBP-Egr, GST-Egr in *E. coli* strain BL21. MBP or GST fusion proteins were purified from bacterial lysates using appropriate affinity column. The GST-Egr protein was purified and used to produce anti-Egr sera in mouse. The antibody was affinity purified by MBP-Egr protein that was affixed to PDVF membrane and eluted using 50 mM glycine pH 2.5. After adjusting to pH 7.0, the buffer was exchanged into phosphate-buffered saline using Amicon Ultracentrifugal Filter Unit with a 10-KDa molecular weight cutoff (Millipore, Etobicoke, Ontario, Canada).

### Immunohistochemistry

Whole-mount eye–brain complexes of third instar larva were prepared as described.^44^ Mouse 24B10 (1 : 200) was from Developmental Studies Hybridoma Bank, at the University of Iowa, Iowa City, IA, USA. Rabbit anti-GFP (1 : 2000) was from Invitrogen (Burlington, Ontario, Canada). Mouse anti-Rac1 (1:200) was from Millipore. The secondary antibodies were Alexa 488-conjugated goat anti-rabbit (1 : 2000), Alexa 594-conjugated goat anti-mouse (1 : 1000) (Molecular Probes, Burlington, Ontario, Canada), and Cy3-conjugated goat anti-mouse (1:1000) (Jackson ImmunoResearch, Burlington, Ontario, Canada). The fluorescent images were captured on Zeiss LSM-710 confocal microscopes (Carl Zeiss Canada Ltd., Toronto, Canada).

## Abbreviations

AP1: activator protein 1
Bsk: Basket
DR: death receptor
Cdc42: cell division control protein 42
ED: eye imaginal disc
Egr: Eiger
FYVE: Fab1, YOTB, Vac1 and EEA1
GEF: guanine nucleotide exchange factor
GFP: green fluorescent protein
GMR: glass multiple promoter
GTPase: guanosine triphosphatase
Mtl: Mig-2-like
JNK: c-Jun N-terminal kinase
LRRK: leucine-rich repeat kinase
MAPK: mitogen activated protein kinase
MF: morphogenetic furrow
NOX: NADPH oxidase
NTR: neurotrophin receptor
PAK1: p21-activated kinase 1
Rab: Ras-related protein
Rac1: Ras-related C3 botulinum toxin substrate 1
RBD: Park1's Rac/Cdc42-binding domain
Rho1: Ras homolog gene family, member 1
RNAi: RNA interference
Sqh: spaghetti squash
dTAB2: *Drosophila* TAK1-binding protein 2
TNF: tumor necrosis factor
TNFR: tumor necrosis factor receptor
dTraf2: *Drosophila* TNF receptor-associated factor 2
TRE: TPA or tetradecanoylphorbol acetate response element
UAS: upstream activating sequence
WT: wild type
